# Echocardiographic estimation of pulmonary hypertension in COVID-19 patients

**DOI:** 10.1007/s12471-022-01702-x

**Published:** 2022-06-30

**Authors:** A. E. P. Wolters, A. J. P. Wolters, T. D. A. van Kraaij, B. L. J. H. Kietselaer

**Affiliations:** 1 Department of Cardiology, Zuyderland Medical Centre, Sittard-Geleen, The Netherlands; 2 Faculty of Health, Medicine and Life Sciences, Maastricht University, Maastricht, The Netherlands; 3 Department of Internal Medicine, Zuyderland Medical Centre, Sittard-Geleen, The Netherlands

**Keywords:** COVID-19, Pulmonary hypertension, Echocardiography

## Abstract

**Introduction:**

Coronavirus disease 2019 (COVID-19) is the cause of a devastating global pandemic and is not likely to be fully resolved in the near future. In most cases COVID-19 presents with mild symptoms, but in a minority of patients respiratory and multi-organ failure may ensue. Previous research has focused on the correlation between COVID-19 and a variety of cardiovascular complications. However, the effect of COVID-19 on pulmonary hypertension (PH) and correlated cardiovascular parameters has not been evaluated extensively.

**Methods:**

This study was designed as a single-centre, semi-quantitative analysis. PH was considered to be present if echocardiographic measurements estimated right ventricular systolic pressure at rest to be 36 mm Hg or higher in combination with indirect indicators of right ventricular overload.

**Results:**

In total, 101 patients (67.3% male) were included in this study, with a mean age of 66 years (range 23–98 years). PH was diagnosed by echocardiographic estimation in 30 patients (29.7%). Echocardiographically estimated PH (eePH) was not correlated with a diagnosis of heart failure or pulmonary embolism. Mortality was significantly higher among COVID-19 patients with eePH (*p* = 0.015). In all 10 of 20 surviving eePH patients in whom echocardiographic follow-up was obtained, echocardiographic estimations of pulmonary pressures showed a significant decrease after a median of 144 ± 72 days.

**Conclusion:**

eePH is frequently observed in COVID-19 patients and is correlated with increased mortality. COVID-19-related eePH appears to be reversible after recovery. Vigilant attention and a low threshold for performance of echocardiography in COVID-19 patients seems warranted, as eePH may be applicable as a prognostic risk factor.

## What’s new?

- In this study, the prevalence of echocardiographically estimated pulmonary hypertension (eePH) in coronavirus disease 2019 (COVID-19) patients was 29.7%.
- Mortality in COVID-19 patients with eePH is significantly higher than in those without. Echocardiographic estimation of right ventricular systolic pressure (RSVP) may be applicable as a prognostic risk factor in COVID-19 patients.
- Pulmonary embolism did not correlate with eePH in COVID-19 patients.
- Estimated RSVP decreased significantly in all patients with eePH following COVID-19 infection on a second echocardiogram after a median of 159 ± 85 days.

## Introduction

In December 2019, first reports appeared on a viral infection that was later identified as severe acute respiratory syndrome coronavirus 2 (SARS-CoV-2), now known as coronavirus disease 2019 (COVID-19) [1]. COVID-19 was declared a global pandemic in March 2020 as a result of its rapid transmission. COVID-19 disease manifestations are subdivided into three stages: an early infectious, a pulmonary and a hyperinflammatory phase. In most cases, patients develop mild symptoms with considerable overlap between these different stages [2]. The most prevailing symptoms include fatigue, dyspnoea, fever and dry cough. However, more severe symptoms may present, including respiratory failure and lung parenchyma degeneration [3]. In a substantial minority of patients, the disease can culminate in multi-organ failure and death [4].

COVID-19 infection has been linked to various cardiovascular complications, including myocardial damage, myocarditis, heart failure, arrhythmias and venous thromboembolism [5–7]. Recent studies show that COVID-19 may cause pulmonary hypertension (PH) and secondary right ventricular dysfunction (RVD) as a result of altered pulmonary haemodynamics and damage to lung parenchyma [6, 8–10]. Provisional data suggest that PH may be present in 13% of COVID-19 patients [5], and in a recent meta-analysis RVD was found in almost 1 out of 5 patients [10]. Possible causes might be pulmonary vasoconstriction, invasive artificial respiration, pulmonary endothelial damage and local inflammatory thrombosis [5, 10]. Patients with RVD showed a threefold higher likelihood of all-cause death, and RVD might be applied in the prognostic risk stratification of COVID-19 patients [10].

PH is defined as a resting right ventricular systolic pressure (RVSP) of 36 mm Hg or higher in the absence of pulmonary valve stenosis [11]. Clinically, PH may present as progressive dyspnoea with fatigue and exhaustion. Physical examination will often show no or only subtle abnormalities. Peripheral and central cyanosis are usually only observable during physical exercise and the typical murmur of systolic tricuspid valve regurgitation or diastolic pulmonary regurgitation is often not apparent. Therefore, symptoms and signs are often non-specific or absent, making PH a difficult clinical diagnosis. Still, early diagnosis and classification of PH are essential in order to determine and initiate an effective treatment [11, 12].

The European COVID-19 vaccination strategy is in progress. By October 2021, 79.7% of the European population had received at least one COVID-19 vaccination [13]. Despite such developments, the COVID-19 pandemic might not be resolved in the near future and recurrent epidemic flare-ups are expected [14]. Therefore, it is of major importance to study factors that could improve the diagnosis and clinical course of COVID-19, as well as factors that determine the prognosis in these patients. The relation between PH and COVID-19 has not been studied extensively. This study aims to estimate the prevalence of PH in COVID-19 patients and to determine correlated clinical parameters, while gaining an impression of its relation to clinical course and prognosis.

## Methods

### Study design and participants

This study was performed at Zuyderland Medical Centre, located in Sittard-Geleen/Heerlen in the Netherlands, from September 2020 until November 2020. During this time, a total of 769 patients were admitted to Zuyderland Medical Centre. This study was designed as a semi-quantitative analysis because of its relatively small population size.

### Inclusion and exclusion criteria

Patients were eligible for inclusion if they had an objectified COVID-19 infection and had undergone echocardiography after diagnosis. We included both inpatients and outpatients. Patients with insufficient echocardiographic imaging, including the absence of a measurable tricuspid regurgitation velocity signal, were excluded. Patients with pulmonary valve stenosis, making it impossible to equalise pulmonary artery pressure (PAP) and RVSP, were also excluded.

### Patient data

Relevant patient data were collected from patients’ electronic health records. These included date of birth, gender, age and comorbidities. Anthropometric data, such as height and weight, were requested before echocardiography based on recent measurements by the patient.

### Diagnosis of COVID-19

The presence of a COVID-19 infection was objectified by a positive COVID-19 polymerase chain reaction test. Previous COVID-19 infections were determined by the presence of SARS-CoV‑2 antibodies in serological tests, measured using SARS-CoV‑2 IgA and IgG ELISA. Patients with an IgA/IgG ratio of 1.1 or higher were considered positive for COVID-19 antibodies.

### Echocardiographic data and PH

PAP was estimated to be equal to RVSP. Right ventricular pressure was estimated using the modified Bernoulli equation by measuring systolic tricuspid valve regurgitation velocity using continuous-wave Doppler signal. Right atrial pressure was estimated based on the diameter and degree of inferior vena cava collapse [11]. In this study, PH was considered to be present if echocardiographic measurements estimated RVSP to be 36 mm Hg. For this estimation we applied standard echocardiographic methods (maximum velocity of tricuspid valve regurgitant flow combined with inferior vena cava width and collapse index), supplemented by indirect indicators of right ventricular overload, such as right ventricular dilatation, right ventricular hypertrophy, reduced right ventricular function and diastolic flattening of the interventricular septum. An increased RVSP combined with one or more indirect indicators of right overload can provide an almost definitive diagnosis of PH [11, 15], termed echocardiographically estimated PH (eePH) in this article. A second echocardiogram was obtained in 10 out of 20 surviving eePH patients.

### Clinical data

Clinical data included the duration of hospitalisation, intensive care hospitalisation, the use of artificial respiration and development of pulmonary embolism (PE) diagnosed using CT angiography. These data were extracted from the patients’ electronic health record. Also included were laboratory values such as C‑reactive protein (CRP), D‑dimers and ferritin.

### Statistical analyses

All statistical analyses were performed using IBM SPSS Statistics 27 (IBM Corp., Armonk, NY, USA). Patient characteristics were measured using independent-samples *t*-test and Fisher’s exact test. Heart valve disease was measured using a chi-squared test. Lastly, the Wilcoxon signed-rank test was used to compare RVSP on the first and second echocardiograms.

## Results

In this study, a total of 101 patients were included. The mean age of the total patient group was 66 years, ranging from 23 to 98 years. In 30 patients, with a mean age 70.9 years, PH was diagnosed on echocardiography (*p* = 0.019). Tab. 1 compares clinical characteristics for patients with and without eePH. For gender and body mass index, no differences were found. Eighty patients (79%) were admitted to the hospital for treatment. A total of 30 patients were admitted to the intensive care unit (ICU); in 16 patients artificial ventilation was required. Artificial ventilation did not significantly impact eePH prevalence. The average hospital stay was 21 days. The average delay between COVID-19 diagnosis and performance of echocardiography was 63 days, 30 days for inpatients and 98 days for outpatients, with a lower average time in eePH patients (*p* = 0.003). The time between COVID-19 diagnosis and echocardiography was significantly lower for eePH inpatients than for inpatients without eePH (*p* = 0.019). The indications for requesting echocardiography did not significantly differ between patients with eePH and those without (Tab. 1). No significant statistical relation was found between admission to hospital or ICU and the presence or absence of eePH. In addition, the total length of hospital stay did not correlate with the presence of eePH, nor did the time between COVID-19 diagnosis and echocardiographic objectification of PH. Mortality in the eePH patient group was significantly higher (33% vs 12.7%, *p* = 0.015).

**Table 1:** Patient characteristics

	Total patient group (*n* = 101)	Pulmonary hypertension^a^ (*n* = 30)	No pulmonary hypertension^a^ (*n* = 71)	*p*-value
Age (years)	65.9 (23–98)	70.9 (23–87)	63.9 (32–98)	**0.019**
Sex (male)	68/101 (67.3%)	19/30 (63.3%)	49/71 (69.0%)	0.645
BMI (kg/m^2^)	27.0 (16–47)	27.1 (16–47)	26.9 (19–38)	0.372
Inpatient	80/101 (79.2%)	24/30 (80%)	56/71 (78.9%)	1.000
Hospital stay (days)	21 (1–104)	18 (1–70)	22 (1–104)	0.476
ICU admission	30/101 (29.7%)	11/30 (36.7%)	19/71 (26.8%)	0.347
Artificial ventilation	16/101 (15.8%)	7/30 (23.3%)	9/71 (12.7%)	0.149
Time between COVID-19 diagnosis and echocardiography (days)	63 (0–196)	39 (0–176)	73 (2–196)	**0.003**
– Inpatient	30 (0–115)	19 (0–100)	38 (2–115)	**0.019**
– Outpatient	98 (2–196)	80 (2–176)	102 (10–196)	0.229
Mortality	19/101 (18.8%)	10/30 (33.3%)	9/71 (12.7%)	**0.015**
Indications for echocardiography				
– (Suspicion of) Heart failure	53/101 (52.5%)	19/30 (63.3%)	34/71 (47.9%)	0.175
– Other/unknown	48/101 (47.5%)	11/30 (36.7%)	37/71 (52.1%)	0.295

*BMI* body mass index, *ICU* intensive care unit

^a^As estimated by echocardiography

**Fig. 1:**
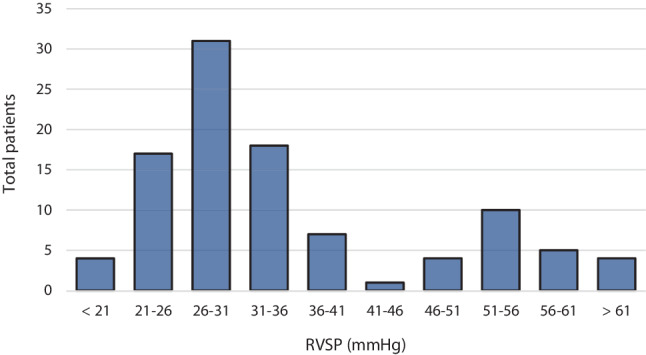
Estimated right ventricular systolic pressure (*RVSP*) in the entire study population

Average estimated RVSP was 34.7 mm Hg, with a mean of 51.1 mm Hg in the eePH group compared to a mean of 27.8 mm Hg in patients without eePH. Fig. 1 presents the distribution of estimated RVSP.

Of all documented comorbidities (PE, pulmonary fibrosis, heart valve disease and heart failure) only heart valve disease showed a significant correlation with the presence of eePH (6 of 30 vs 0 of 71 patients, *p* < 0.001). None of the COVID-19-associated laboratory tests (D-dimer, CRP, ferritin) provided results that differed significantly between the two groups (Tab. 2).

**Table 2:** Prevalence of pulmonary hypertension and comorbidity, raised laboratory values

	Total patient group	Pulmonary hypertension^a^	No pulmonary hypertension^a^	*p*-value
Pulmonary embolism	20/79 (25.3%)	4/23 (17.4%)	16/56 (28.6%)	0.796
Lung fibrosis/COPD	18/101 (17.8%)	7/30 (23.3%)	11/71 (15.5%)	0.398
Heart valve disease	6/101 (5.9%)	6/30 (20.0%)	0/71 (0%)	*<* *0.001*
Heart failure	6/101 (5.9%)	3/30 (10.0%)	3/71 (4.2%)	0.358
Raised D‑dimer	38/65 (58.5%)	14/19 (73.7%)	24/46 (52.2%)	0.167
Raised CRP	71/78 (91.0%)	22/23 (95.7%)	49/55 (89.1%)	0.667
Raised ferritin	58/77 (75.3%)	15/23 (65.2%)	43/54 (79.6%)	0.248

*COPD* chronic obstructive pulmonary disease, *CRP* C-reactive protein

^a^As estimated by echocardiography

**Fig. 2:**
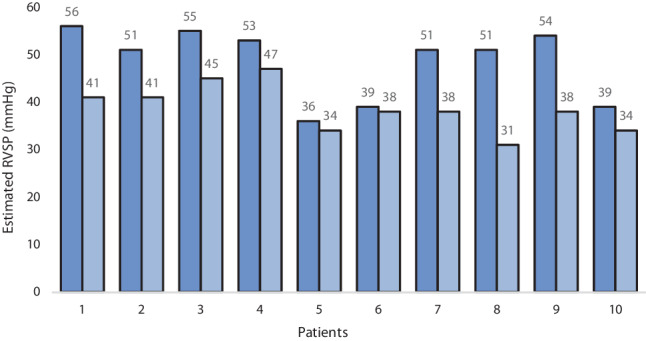
Mean estimated right ventricular systolic pressure (*RVSP*) on first and second echocardiogram in individual patients with pulmonary hypertension estimated by echocardiography

Mean estimated RVSP on the first echocardiogram was 49 ± 7 mm Hg, whereas mean estimated RVSP was 39 ± 5 mm Hg on the second echocardiogram after 159 ± 85 days. The mean decrease in RVSP on the second echocardiogram obtained in 10 out of 20 surviving eePH patients was 10 ± 6 mm Hg (*p* = 0.005) (Fig. 2).

## Discussion

We report on eePH in 29.7% of patients with COVID-19 infections, most of whom were admitted to hospital. A diagnosis of PH based on echocardiography was clearly correlated with higher mortality, underlining the importance of this procedure. Surprisingly, the presence of eePH did not correlate with (previous) PE or the presence of heart failure or artificial ventilation, and was not associated with higher values for COVID-19-related laboratory parameters. Only valvular heart disease was related to the presence of eePH. In all surviving patients, in whom echocardiographic follow-up was obtained, estimated RVSP decreased during the following months, suggesting a partly reversible pathophysiology.

Previous studies have suggested a correlation between COVID-19 and PH; however, little is known about its exact prevalence. Pagnesi et al. [5] reported a PH prevalence of 12.0% in a group of hospitalised COVID-19 patients in Italy. Our data suggest a higher prevalence, which may be due to the inclusion of patients admitted to the ICU, accounting for more than a third of the patients diagnosed with PH. In addition, Pagnesi et al. described a correlation between the incidence of PE and PH. It has been suggested that PE might be a factor in the pathophysiology of PH in patients with COVID-19 [16]. In the present patient cohort, no significant correlation was found between eePH and PE. We speculate that the diagnostic workup of PE in patients with COVID-19, especially early in the pandemic, was not as thorough due to the strain on the care systems and widespread D‑dimer elevation in COVID-19 patients, possibly causing underestimation of the prevalence of PE in patients with PH. Furthermore, COVID-induced widespread angio-inflammatory changes throughout the lung parenchyma may provoke PH in the absence of PE [9]. On the other hand, the correlation of eePH with valvular heart disease in our cohort does suggest a cardiac factor predisposing COVID-19 patients to development of PH, making a multifactorial aetiology the most plausible explanation.

During follow-up of patients with possible COVID-19-related eePH, a second echocardiogram showed a significant decrease in estimated RVSP after a median of 144 ± 72 days. This suggests that the prognosis of eePH itself, while clearly related to increased mortality, is generally positive in the long term, with the possibility for full recovery. This statement is supported by our data suggesting a higher prevalence of eePH in patients with a shorter delay between COVID-19 diagnosis and echocardiography. The importance of PH as a prognostic factor for mortality was also highlighted by a recent meta-analysis [10]. To the best of our knowledge, these are the first preliminary data on the follow-up of eePH in COVID-19 patients. However, due to the small and non-selected sample size of patients undergoing repeat echocardiography, our data should be considered preliminary and interpreted with caution.

One of the limitations of this study was the fact that in most patients the first echocardiogram was made during or after a COVID-19 infection. Therefore, no data on pre-existing PH within our patient group could be acquired. A further limitation is the selection of patients that underwent echocardiography, including both inpatients and outpatients. Due to the risk of disease spreading by performing echocardiography, many patients with a clinical suspicion of PH or heart failure may not have had an indication for echocardiography. This may have resulted in underestimation of the prevalence of eePH among COVID-19 patients. The long delay between COVID-19 diagnosis and performance of echocardiography in our study may also point to physician unawareness concerning the correlation with PH and resulting underdiagnosis of PH in COVID-19 patients. Finally, our study is severely limited by its scope and size.

In conclusion, PH was diagnosed on echocardiography in 29.7% of this unselected group of COVID-19 patients. These eePH patients showed significantly higher mortality than patients without eePH. Furthermore, our data suggest COVID-19-related eePH might be reversible in the long term. These results need to be confirmed by further research. Given the high mortality rate associated with eePH, we suggest vigilant attention to relevant symptoms and a low threshold for echocardiography in COVID-19 patients, with special attention to the possible detection of PH.
